# Increasing CRISPR/Cas9-mediated gene editing efficiency in T7 phage by reducing the escape rate based on insight into the survival mechanism: T7 Phage escape in CRISPR/Cas9: strategies for efficient editing

**DOI:** 10.3724/abbs.2024030

**Published:** 2024-05-16

**Authors:** Mingjun Sun, Jie Gao, Hongjie Tang, Ting Wu, Qinqin Ma, Suyi Zhang, Yong Zuo, Qi Li

**Affiliations:** 1 College of Life Sciences Sichuan Normal University Chengdu 610101 China; 2 Luzhou Laojiao Co Ltd Luzhou 646000 China; 3 National Engineering Research Center of Solid-State Brewing Luzhou 646000 China

**Keywords:** Key words: CRISPR, Cas9, T7 phage, gene editing, escape

## Abstract

Bacteriophages have been used across various fields, and the utilization of CRISPR/Cas-based genome editing technology can accelerate the research and applications of bacteriophages. However, some bacteriophages can escape from the cleavage of Cas protein, such as Cas9, and decrease the efficiency of genome editing. This study focuses on the bacteriophage T7, which is widely utilized but whose mechanism of evading the cleavage of CRISPR/Cas9 has not been elucidated. First, we test the escape rates of T7 phage at different cleavage sites, ranging from 10 ^-2^ to 10 ^-5^. The sequencing results show that DNA point mutations and microhomology-mediated end joining (MMEJ) at the target sites are the main causes. Next, we indicate the existence of the hotspot DNA region of MMEJ and successfully reduce MMEJ events by designing targeted sites that bypass the hotspot DNA region. Moreover, we also knock out the ATP-dependent DNA ligase *1*. *3* gene, which may be involved in the MMEJ event, and the frequency of MMEJ at *4*. *3* is reduced from 83% to 18%. Finally, the genome editing efficiency in T7 Δ *1*. *3* increases from 20% to 100%. This study reveals the mechanism of T7 phage evasion from the cleavage of CRISPR/Cas9 and demonstrates that the special design of editing sites or the deletion of key gene *1*. *3* can reduce MMEJ events and enhance gene editing efficiency. These findings will contribute to advancing CRISPR/Cas-based tools for efficient genome editing in phages and provide a theoretical foundation for the broader application of phages.

## Introduction

Bacteriophages have a wide range of applications, such as vectors for vaccines, gene therapy, and combating antibiotic-resistant bacteria [ 1‒ 4]. An ideal phage used in the above situations should have a series of characteristics, such as long-term stability, scalable production, efficient recognition and killing of bacterial pathogens specifically. Achieving these characteristics of bacteriophages requires an effective genomic editing tool, and some cases of successful applications of gene editing modified phages for personalized treatments of *Acinetobacter baumannii*, *Pseudomonas aeruginosa*, and *Mycobacterium abscessus* infections have been reported [ 5‒ 7]. Therefore, the development of gene editing technology for bacteriophages can facilitate their application.

CRISPR/Cas is an adaptive immune system discovered in bacteria and archaea, and 6 types of CRISPR/Cas systems have been identified. Several CRISPR/Cas-based genome editing methods have been reported for bacteriophages, including the CRISPR/Cas9 system (type II), the CRISPR/Cas3 system (type I), and the CRISPR/Cas13a system (type VI) [ 8‒ 13]. However, in practical operation, bacteriophages can escape the cleavage of CRISPR/Cas, and the escaped phages are called escapers. The escaper not only reduces the efficiency of bacteriophage genome editing systems but also increases the difficulty of screening positive phages [ 8‒ 11, 14]. Thus, understanding the escape mechanism of bacteriophages from the cleavage of CRISPR/Cas can advance the development of efficient genome editing tools and accelerate the construction of bacteriophages for therapeutic purposes.

Some studies have reported the mechanism by which bacteriophages escape from CRISPR/Cas. First, certain bacteriophages can express anti-CRISPR (Acr) proteins, rendering and leading to the occurrence of escapers [ 15‒ 18]. Most Acrs can interact with Cas proteins, which results in eliminating the DNA cleavage or binding capabilities of Cas proteins [19]. Acrs in bacteriophages have been identified for several types of CRISPR/Cas systems, including type I (AcrIC1 present in *Moraxella bovoculi* prophage), type II (AcrIIA1 present in *Listeria monocytogenes* prophage), type III (AcrIIIB1 present in *Sulfolobus* virus), and type V (AcrVA1 present in *M*. *bovoculi* prophage) [ 20‒ 24]. Second, bacteriophages can also evade the cleavage of Cas protein by modifying their own genomes. For instance, the P22 phage can modify its genome by utilizing methyltransferases from its host *Salmonella*. In this kind of escaper, it is possible to delete the methyltransferase gene of the host bacteria to reduce the escape phenomenon [ 18, 25, 26]. Furthermore, some *Bacillus* phages can also produce methyltransferases by themselves [27]. In addition to methylation, bacteriophages can also cause modifications such as hydroxy methylation, glycosylation, and N-acetylation [ 28, 29]. Moreover, a large portion of escaped phages are attributed to mutations occurring in the bacteriophage genome under the cleavage pressure of the Cas protein [ 30‒ 33]. For instance, microhomology-mediated end joining (MMEJ) has been observed under the pressure of cleavage of the genome in the bacteriophage T4. The occurrence of MMEJ relies on the pair of small identical homologous sequences on both sides of the target site. Typically, the repeat sequence is shorter than 15 bp. After the target site has been cleaved, the fragment between the two repeat sequences is lost by recombination, which causes the repaired bacteriophage to escape cleavage by Cas proteins. It was later discovered that the MMEJ is attributed to the recombinase gene *uvsX* in the bacteriophage T4 [34]. In summary, recent research on the mechanism by which bacteriophages escape from the cleavage of CRISPR/Cas has been limited to bacteriophages such as *Pseudomonas aeruginosa* prophages, *Bacillus* phages, and T4 phages.

T7 phage has been widely applied in various fields [ 3, 4, 35]. Specifically, this phage lacks Acrs and genome modification systems. The mechanism by which T7 phage escapes from cleavage of CRISPR/Cas has not been reported. Here, the CRISPR/Cas9-based genome editing system pEcCas/pEcgRNA established in our previous work was used to study the escape of the T7 phage [36].

In this study, we revealed that T7 phage escapes the cleavage of the CRISPR/Cas system through DNA point mutations and deletions at targeted sites, and this deletion pattern is consistent with that of MMEJ previously observed in the T4 phage. A hotspot region was observed for MMEJ events, the guide RNA (gRNA) designed to avoid these hotspot DNA regions will decrease the escape rate and increase the corresponding genome editing efficiency. Based on the analysis of the genome, ATP-dependent DNA ligase gene *1*. *3*, which may be involved in the MMEJ process, was focused on. Subsequently, this gene was deleted, reducing the escape rate and increasing the efficiency of genome editing based on the escape mechanism. The discovery in this study can accelerate the research and application of T7 phage and can also serve as a reference for studying the escape mechanism in other bacteriophages.

## Materials and Methods

### Bacterial strains and culture methods

*E*. *coli* DH5α and MG1655 used in this study were cultured in LB media at 37°C. The antibiotics kanamycin (50 μg/mL) and spectinomycin (50 μg/mL) were added to the LB media if necessary. Strains were stored at –80°C with a final concentration of 25% glycerol. For strain recovery, cultures were streaked on LB agar plates, and isolated colonies were inoculated into LB broth. The T7 phage was cultured in the logarithmic growth phase of *E*. *coli* MG1655. All strains are listed in Supplementary Table S1.

### Reagents and enzymes

The restriction endonucleases used in this study were purchased from Thermo Fisher Scientific (Waltham, USA). DNA polymerases, including 2×Phanta Flash Master Mix (Vazyme Biotechnology Co., Ltd., Nanjing, China) for high-fidelity DNA amplification and 2×Es Taq MasterMix (Dye) (Jiangsu Cowin Biotech Co., Ltd., Taizhou, China) for colony PCR, were used. A reagent kit for plasmid extraction was obtained from Tiangen (Tiangen Biotech Co., Ltd., Beijing, China), and a reagent for DNA purification was obtained from TransGen (Beijing, China) following the manufacturer’s instructions. A ClonExpress One Step Cloning Kit (Vazyme Biotechnology Co., Ltd.) was used for the assembly of plasmids.

### Plasmids construction

*E*. *coli* DH5α was used for the maintenance and construction of plasmids. All plasmids and primers employed in this study are detailed in Supplementary Tables S1 and S2. The plasmid pEcCas (Addgene, Watertown, USA) originated from our previous research [36]. The pEcgRNA-X series plasmids, targeting specific genes as denoted by ″X″, were constructed following the methods outlined in our publications [36]. For the construction of plasmids in the pEcgRNA-X-donor series, the plasmid backbone was initially amplified with the primers gRNAdonor-up/gRNAdonor-dn, and after linearization, it was assembled with donor DNA carrying the repair templates (amplified from the T7 genome, carrying 0.5 kb upstream and 0.5 kb downstream homologous arms) using the ClonExpress One Step Cloning Kit (Vazyme Biotechnology Co.).

### Plasmids transformation and escape rate testing

The plasmids pEcCas and pEcgRNA-X were transformed into *E*. *coli* MG1655 as experimental group strains. *E*. *coli* MG1655 containing the pEcCas plasmid, in which the gRNA does not target any loci in the T7 genome, was used as the control group strain. For the escape rate test, 100 μL of bacteriophage (10 ^3^‒10 ^8^ plaque forming units (PFUs)/mL) was diluted in LB medium, and then mixed with 300 μL of logarithmic phase experimental group *E*. *coli* or control group *E*. *coli* cells (10 ^8^ colony forming units (CFUs)/mL). Next, the mixtures were mixed with 5.0 mL of semi-solid LB medium after being incubated at 37°C for 5 min. The resulting mixture was poured onto pre-made plates with a solid LB agar layer at the bottom. The plates were then incubated overnight at 37°C. Subsequently, the PFU of bacteriophages on the plates was counted, and the escape rate was calculated.

The escape rate= the number of PFUs of the experimental group/the number of PFUs of the control group.

### Sequencing of the targeted gene of the escaped T7 phage

Under sterile conditions, individual escaped phage plaques were picked into 20 μL of ddH _2_O. The escaped phage plaques in the PCR tubes were allowed to incubate at room temperature for 1 h, with intermittent stirring every few minutes to ensure the complete release of phage particles into ddH _2_O, and 1 μL was used as the template for PCR amplification. The primers were designed at 500 bp upstream and downstream of the targeted gene *5* (2115 bp), *1.3* (1080 bp), *1.7* (591 bp), *1.2* (258 bp), and 200 bp upstream and downstream of *4.3* (213 bp). For efficient release of the phage genome during PCR, the predenaturation time was extended to 10 minutes at 95°C. Following purification and gel electrophoresis identification of the PCR amplification products, the DNA was excised and purified from the gel for subsequent sequencing.

### Genome editing of T7 bacteriophages

Initially, the corresponding pEcgRNA-X-donor plasmids were transformed into *E*. *coli* cells along with the pEcCas plasmid. Next, 300 μL of logarithmic-phase *E*. *coli* cells induced with 10 mM arabinose was mixed with 10 ^4^~10 ^5^ PFU of bacteriophage, and the mixture was incubated at 37°C for 5 min. Next, the mixture was combined with 5.0 mL of LB medium, poured onto pre-made LB agar plates, and incubated overnight at 37°C. Next, bacteriophage PCR was performed using the primers 1.3-KO-verf-up/1.3-KO-verf-dn, 4.3-KO-verf-up/4.3-KO-verf-dn, and 1.7-KO-verf-up/1.7-KO-verf-dn for the validation of individual phage plaques. PCR amplicons were subjected to DNA sequencing.

## Results

### DNA point mutations and deletions are the main reasons for the T7 phage escaping from the cleavage of CRISPR/Cas9

To explore the mechanism by which different targeted genes within the T7 phage under the cleavage of CRISPR/Cas9, one essential gene (gene *5*) and three non-essential genes (gene *1*. *3*, *1*. *7*, and *1*. *2*) were selected as targeted sites. Four gRNAs targeting these genes along with the control plasmid pEcgRNA-Null, which does not target any gene loci in the T7 genome, were transformed into *E*. *coli* MG1655, which constitutively expresses the Cas9 plasmid pEcCas9. Infections were performed using T7 phage on the forementioned *E*. *coli* strains carrying both pEcCas and the corresponding pEcgRNA plasmids. The escape rate of the T7 phage was calculated on the next day.

**Figure 1:**
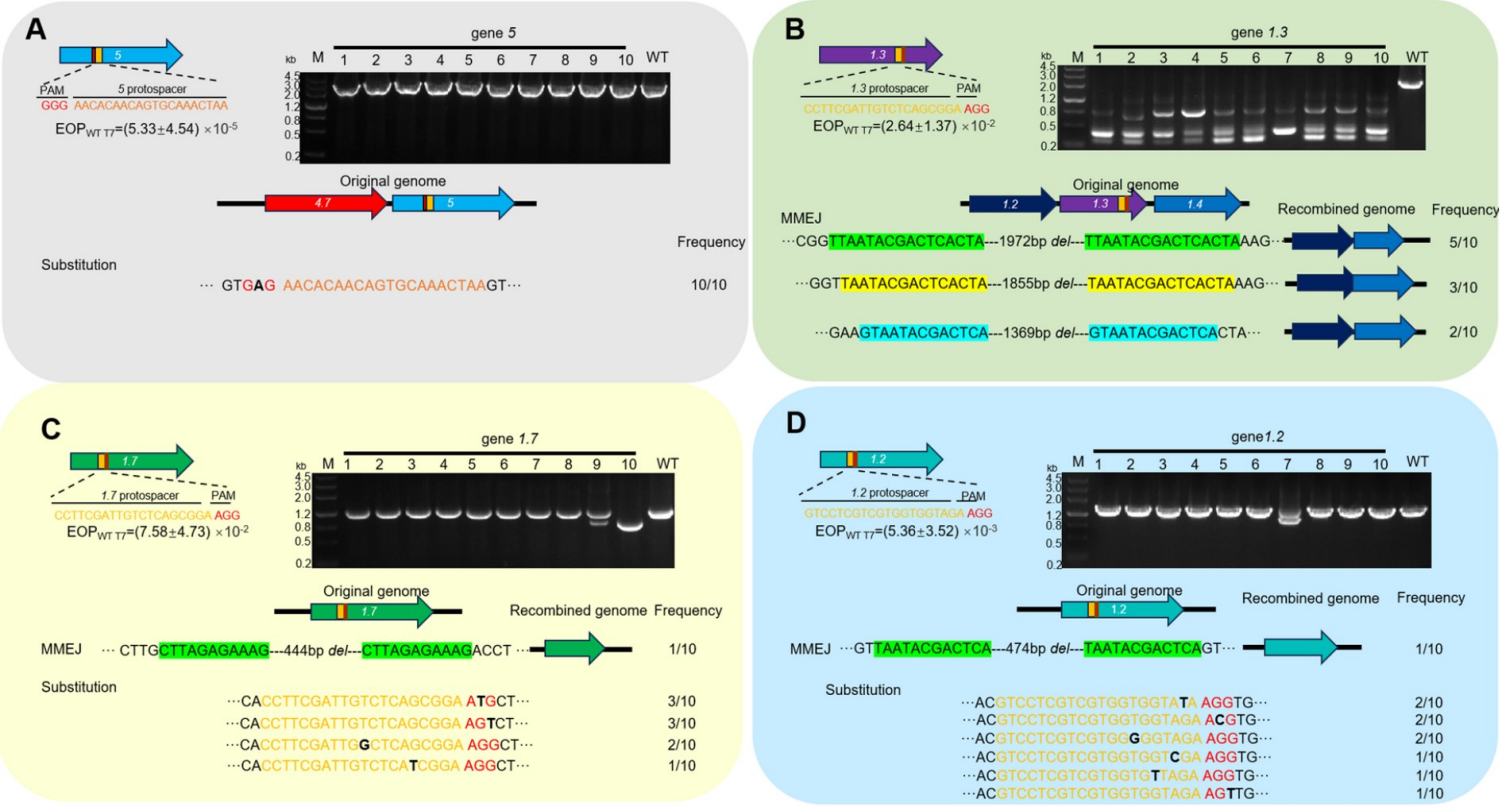
Figure 1 Analysis of the mechanisms by which the T7 phage escapes the cleavage of CRISPR/Cas9 (A) Information of the gene 5 targeted site and the reason for the escaped phage at gene 5 site. (B) Information of the 1.3 targeted site and the reason for the escaped phage at 1.3 site. (C) Information of the 1.7 targeted site and the reason for the escaped phage at 1.7 site. (D) Information of the 1.2 targeted site and the reason for the escaped phage at 1.2 site. “WT” represents the wild type.

The escape rate of essential gene *5* was approximately 10 ^–6^ ( Figure 1A). The escape rates for the non-essential genes *1*. *3* and *1*. *7* were approximately 10 ^–2^, while in gene *1*. *2*, the escape rate was approximately 10 ^–3^ ( Figure 1B–D). In comparison, the escape rates of non-essential genes were higher than those of essential genes. After collection of the surviving plaques, primers were designed to target the 500 bp of upstream and downstream of the targeted site. Following PCR amplification, the size of the products was analyzed and it was shown that some DNA fragments amplified from the escaped phage were smaller in size with the wild type, suggesting that the T7 phage may have deleted DNA fragments near the target site through a repair mechanism. This phenomenon was observed in almost all non-essential genes ( Figure 1B–D), while no deletion was observed in the essential gene *5*.

Next, we sequenced the amplified DNA fragments obtained above. The results showed that there were primarily two types of mutations. For essential gene *5*, all the mutations manifested as point mutations in the protospacer adjacent motif (PAM) region, and they were synonymous mutations (corresponding codon changes from ACC to ACT, both encoding serine) ( Figure 1A). And for non-essential genes, the target sequence and the PAM region were deleted at different frequencies ( Figure 1B–D). For these deleted sequences, a comparative analysis with the parental phage genome was carried out. In the parental phage, a segment with a short repeat sequence approximately 8 to 15 bp in length was present on both sides of the deleted region. Obviously, the target sequence and the PAM region were deleted by the recombination of the repeat sequence. This recombination pattern was the same as the MMEJ previously observed in the T4 phage [34]. Thus, DNA point mutations and deletions at the target gene loci are the main causes of T7 phage escape from the cleavage of CRISPR/Cas9, and the deletion occurs through MMEJ.

### Avoiding the hotspots DNA region for MMEJ can reduce the escape rate and improve the efficiency of gene editing

**Figure 2:**
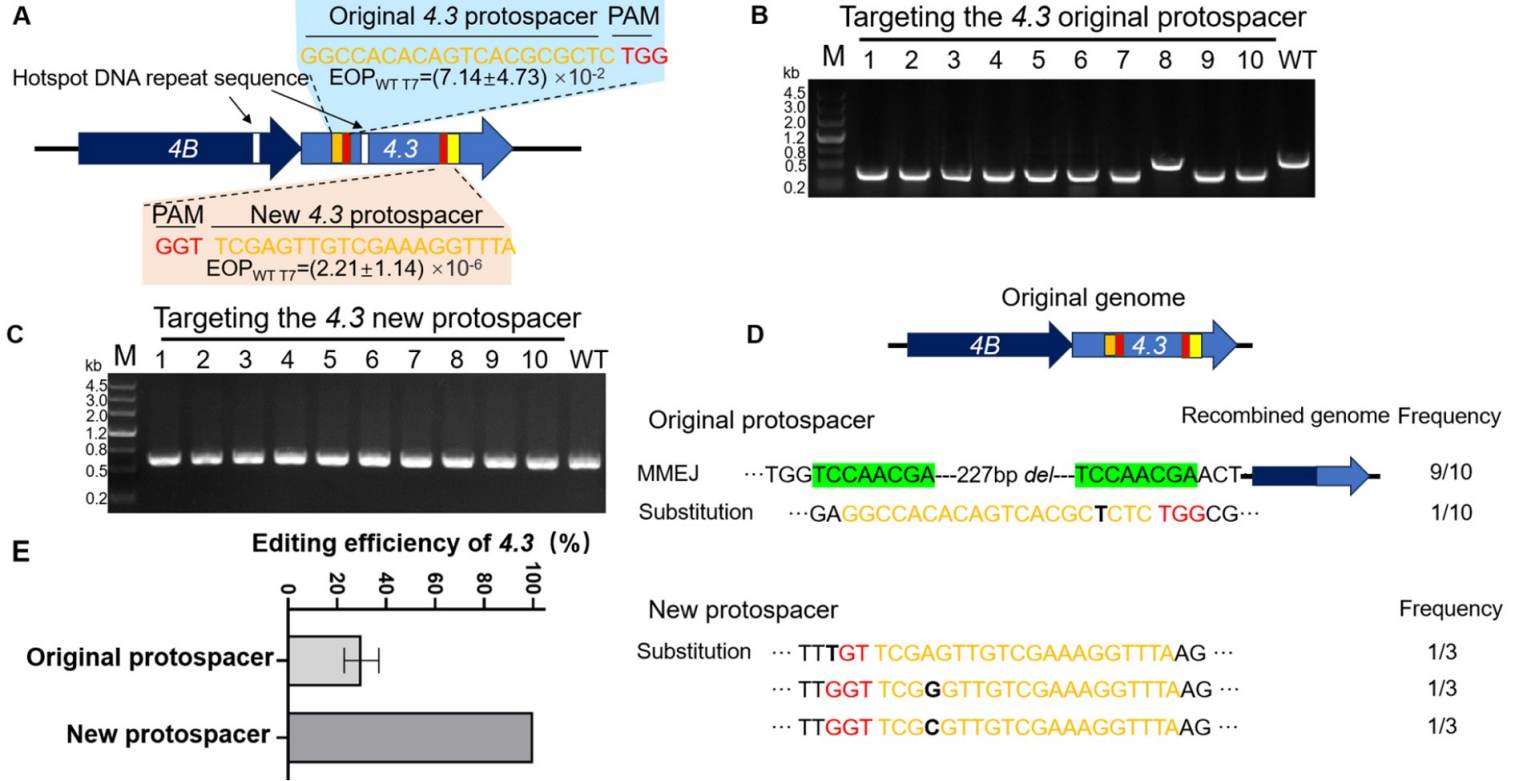
Figure 2 The hotspot DNA region existed for MMEJ events, and the design of targeted sites bypassing the hotspot DNA region can reduce MMEJ events and increase the efficiency of gene deletion (A) The information of the 4.3 targeted sites including original 4.3 protospacer and new 4.3 protospacer; the hotspot DNA region is marked in white. (B) PCR amplification results of escaped plaques under the targeting of the original 4.3 site. (C) PCR amplification results of single escaped plaques under the targeting of the new 4.3 site. (D) DNA Mutation type of 4.3 by targeting the original site and the new site. (E) Gene deletion efficiency of 4.3 by targeting the original site and the new site. “WT” represents the wild type.

An online analysis tool was utilized to analyze the genomic information of the T7 genome, and multiple potential repeat sequences for MMEJ were found to exist in almost all genes [37]. We were curious about whether there is a difference in the frequency of MMEJ among different repeat sequences at the same cleavage site. Therefore, the non-essential gene *4*. *3*, which carries 16 pairs of repeat sequences, was selected as a test site. The cleavage site was designed between all repeat sequences, and the plasmid pEcgRNA-T7-4.3-original was constructed to test the escape type in the T7 phage ( Figure 2A). Ten escape mutants were detected after the cleavage of the *4*. *3* gene, and 9 escapers survived through MMEJ with the repeat sequence ″TCCAACGA″, while the remaining one involved point mutations in the PAM region. No second type of MMEJ was observed in these escape mutants, indicating a hotspot for MMEJ occurrence at this site ( Figure 2B). These results suggested that if the cleavage site is designed between all repeat sequences, there will be a hotspot DNA region for MMEJ in the escaped phage.

To determine whether cutting outside the hotspot region of repeated sequences could reduce the frequency of MMEJ, a new gRNA plasmid named pEcgRNA-T7-4.3-new was designed to target the gene *4*. *3* outside the MMEJ hotspot region ( Figure 2A). Subsequently, the escape rate was determined on T7 phage using the new gRNA plasmid. Fortunately, the escape rate decreased from 7.1×10 ^–2^ to 2.2×10 ^–6^ when the new spacer sequence was used ( Figure 2A). Next, survived plaques under the cleavage of CRISPR/Cas9 were collected, primers were designed to target the 200 bp of upstream and downstream of the targeted site, and the PCR-amplified products were analyzed. The results showed that the MMEJ disappeared in all escaped phage, and the sizes of the PCR-amplified products in all escaped phage were consistent with those in the wild type. ( Figure 2C). Subsequently, three bands were randomly selected for sequencing. The results showed that the escape type was all attributed to mutations in the target sequence or the PAM region ( Figure 2D and Supplementary Figure S1).

Moreover, we sought to explore whether the different MMEJ rates could impact the gene editing efficiency of the T7 phage. Thus, donor DNA capable of deleting the *4*. *3* gene was constructed. These two plasmids were transformed into *E*. *coli* MG1655 containing the pEcCas, and then T7 was used to infect them respectively. The results showed that the editing efficiency of the T7 phage using the pEcgRNA-T7-4.3-original-donor was only 20%, while that using the pEcgRNA-T7-4.3-new-donor was 100% ( Figure 2E and Supplementary Figure S4A,B). This result indicated that reducing the MMEJ rate can enhance the editing efficiency of the T7 phage. Another gene, *4*. *2*, was tested to confirm our conclusion ( Supplementary Figure S2).

### The frequency of MMEJ can also be reduced by the deletion of the *1.3* gene in the T7 phage

**Figure 3:**
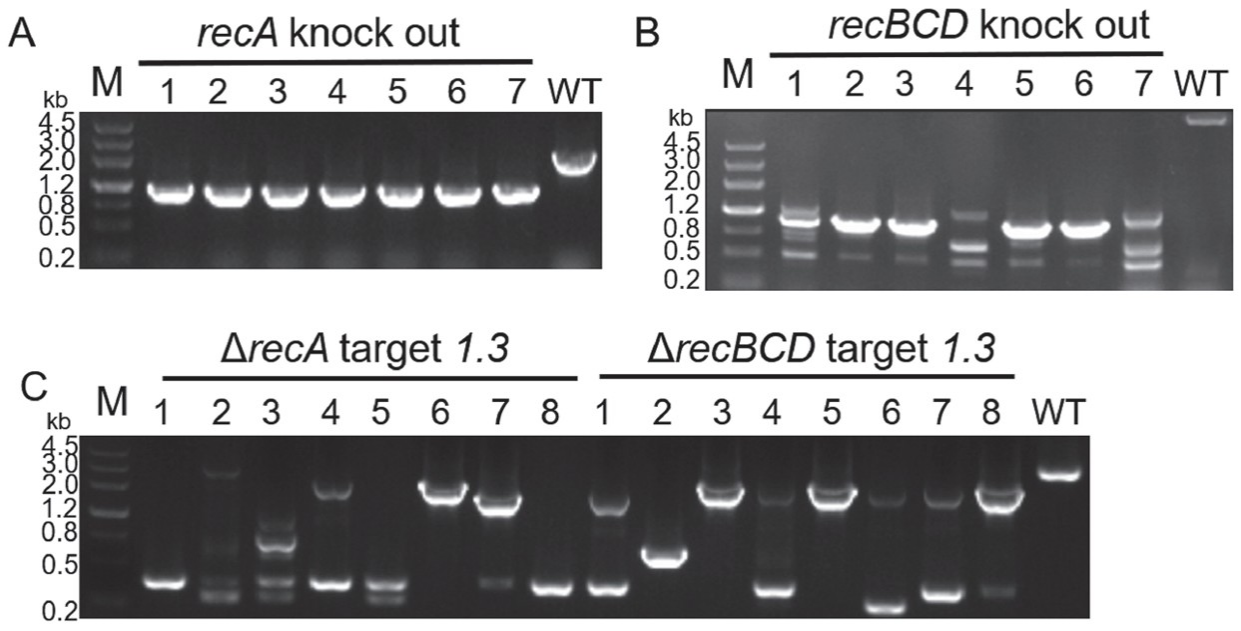
Figure 3 Results of *recA* and *recBCD* gene knockout in *E*. *coli* MG1655, and the escape types of T7 phage targeted *1*. *3* gene in the above two mutant hosts (A) Results of recA gene deletion in E. coli MG1655 showed that colonies 1-7 were strains in which recA was successfully deleted. Sample No 5 was randomly selected for subsequent experiments. (B) Results of recBCD gene deletion in E. coli MG1655 showed that colonies 2, 3, 5, and 6 were strains in which recBCD was successfully deleted. Sample No 6 was randomly selected for subsequent experiments. (C) Results of DNA amplification of the 1.3 gene under Cas9 cleavage in ΔrecA and ΔrecBCD MG1655, showing DNA deletions indicated by the sizes of the PCR products. Sequencing of bands of the same size revealed that MMEJ was present in all 8 escape mutants in each group.

To investigate which gene is involved in the occurrence of MMEJ in the T7 phage, we first focused on the genes in *E*. *coli*. RecA combines with ssDNA to form stable nucleoprotein filaments, which are crucial for guiding DNA strand exchange and pairing homologous sequences. Additionally, RecBCD, acting as an exonuclease, plays a critical role in the MMEJ process in *E*. *coli*. Thus, we hypothesized that the expression of *recA* and *recBCD* in *E*. *coli* might be involved in the MMEJ process of the T7 phage. Next, these genes were knocked out in *E*. *coli* MG1655, and the resulting deletion strains were used to retest escape tests with T7 phage ( Figure 3A,B). However, the results showed that the deletion of these genes in *E*. *coli* MG1655 did not reduce the escape rate of the T7 phage and that the frequency of MMEJ did not decrease ( Figure 3C). Thus, we concluded that the occurrence of MMEJ in the T7 phage is not related to the genes of *E*. *coli*.

**Figure 4:**
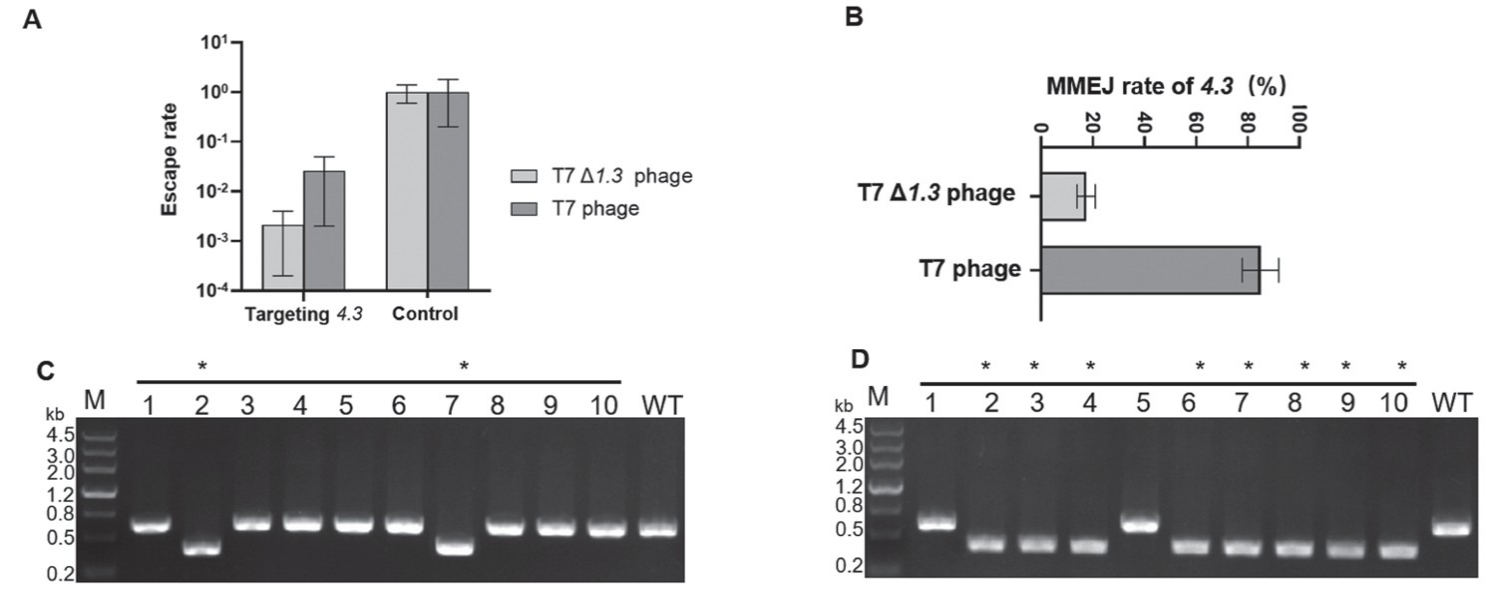
Figure 4 The frequency of MMEJ can be reduced by the deletion of the *1*. *3* (A) Escape rates at the 4.3 gene in T7 Δ1.3 and T7 wild type. (B) MMEJ rate in 4.3 was reduced by the deletion of 1.3 gene in T7 wild type. (C) The PCR amplification results of 4.3 site in T7 Δ1.3. (D) The PCR amplification results of 4.3 site in T7 wild type. “control” represents the control group (MG1655 with nontarget gRNA), and “*” represents the MMEJ events. “WT” represents the wild type.

Next, we focused on the T7 phage itself, and the genomic information of the T7 phage was further analyzed. First, for MMEJ in the T4 phage, MMEJ has been reported to occur in T4 phage-dependent recombinant enzymes [34]. However, there were no recombinant enzymes in the T7 phage. Thus, the presence of MMEJ in *E*. *coli* was analyzed, and this ligase played an essential role in MMEJ [38]. Thus, we selected the only ligase gene, *1*. *3*, in the T7 phage as a research object. Next, this gene was knocked out in the T7 phage ( Supplementary Figure S3A), and this mutated phage was used for escape tests at the *4*. *3* original site. The results showed that the escape rate decreased from 7.1×10 ^–2^ to 4.29×10 ^–3^ ( Figure 4A). Next, the targeted genes of 11 selected survived T7 phage plaques were amplified, the size of the DNA was analyzed, and the amplified products were sequenced immediately. The results showed that in the wild-type T7 phage, 9 of 11 escapers showed different sizes from their parental phage, but only 3 of 11 in the T7 phage Δ *1*. *3*. The sequencing results also demonstrated that MMEJ events occurred in all amplified products with different sizes ( Figure 4C,D). Furthermore, we selected 20 more survived plaques and determined their escape types. The results showed that in the targeted site *4*. *3*, the MMEJ rate of wild-type T7 phage was approximately 83% in this targeted site ( Figure 4B). In contrast, the MMEJ rate of T7 phage Δ *1*. *3* was only about 18%, and most escapers carried point mutations in the targeted site or the PAM region ( Figure 4B and Supplementary Figure S3B). Therefore, it was demonstrated that the deletion of *1*. *3* gene in the T7 phage can reduce the frequency of MMEJ.

### The gene editing efficiency of the T7 phage can be improved by the deletion of the *1.3* gene

**Figure 5:**
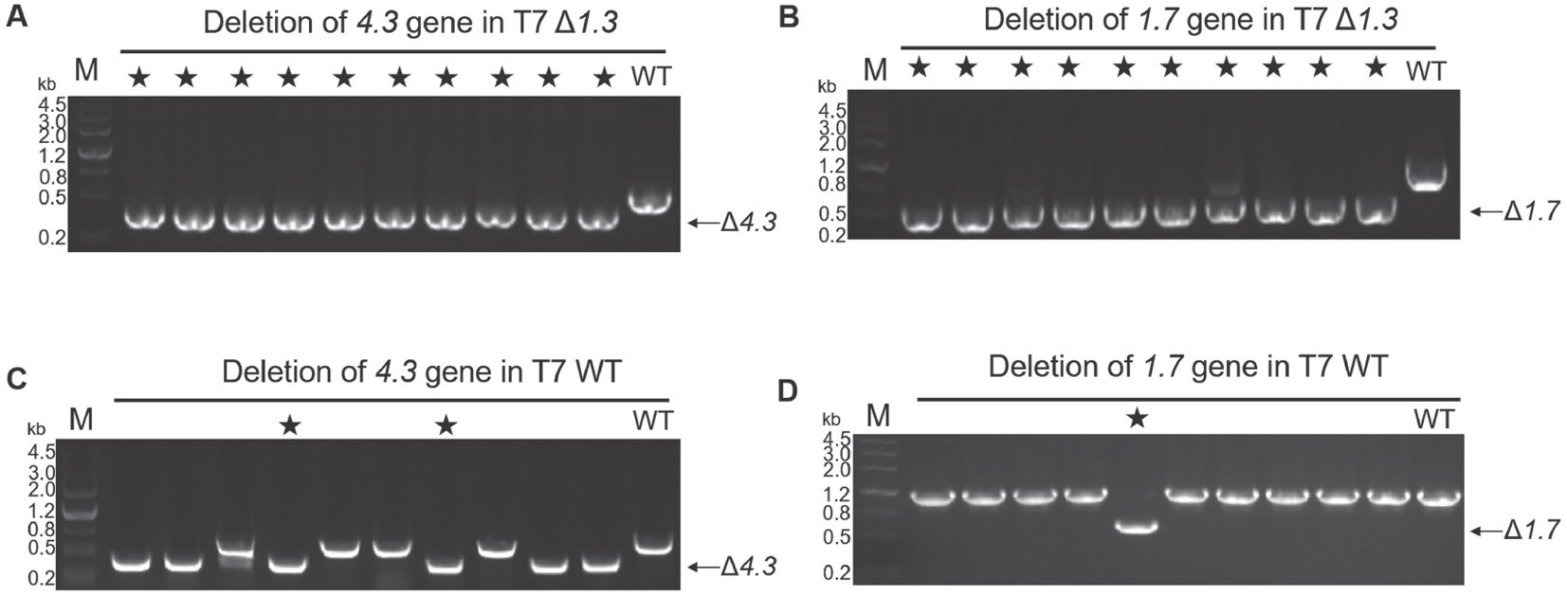
Figure 5 The gene deletion efficiency in T7 Δ *1*. *3* and T7 wild type (A) The deletion of 4.3 in T7 Δ1.3. (B) The deletion of 1.7 in T7 Δ1.3. (C) The deletion of 4.3 in T7 WT. (D) The deletion of 1.7 in T7 WT. “★” represents successful deletion. “WT” represents the wild type.

After demonstrating that the deletion of the *1*. *3* gene in the T7 phage can reduce the frequency of MMEJ, we expected that the editing efficiency could be improved by reducing the escape rate. Therefore, we tested the gene deletion efficiency of two genes, *4*. *3* and *1*. *7*, in T7 Δ *1*. *3*, while wild-type T7 was used as a control. The results showed that the gene editing efficiency of T7 Δ *1*. *3* in both genes was 100% ( Figure 5A,B), while in wild-type T7, it was only 20% ( Figure 5C,D). Notably, although some DNA bands in wild-type T7 were similar in size after *4*. *3* deletion, the sequencing results showed that only two bands of them have the deletion of *4*. *3*, while the remaining bands are the results of MMEJ ( Supplementary Figure S4C,E). The sequencing results of all the DNA bands can be found in the supplementary files ( Supplementary Figure S3). Here, the results demonstrated that the deletion of *1*. *3* in T7 can indeed improve the efficiency of T7 gene editing.

## Discussion

This study revealed that the escape mechanisms of T7 phage under the cleavage of CRISPR/Cas9 involve DNA point mutations and deletions in the targeted genes. DNA point mutations were observed in all tested genes, while MMEJ occurred specifically in non-essential genes. Both mechanisms can result in T7 phage escape from the cleavage of CRISPR-Cas9, thereby reducing the efficiency of editing the genome of T7. For DNA point mutations, compared with those in previous studies, the types of DNA mutations are different. In the T7 phage, point mutations primarily involve DNA base substitutions and synonymous mutations, whereas in the *Listeria* phage LP-125 and T4 phage, nonsense mutations and DNA base additions and deletions are more frequent [ 34, 39, 40]. Notably, it was observed that the frequency of synonymous mutations reached 100% in the T7 phage when essential gene *5* was targeted ( Figure 1A). For DNA deletions, the T7 phage utilizes a mechanism called MMEJ, which requires only very short homologous sequences (8–15 bp) to recombine its cleaved genome. In contrast, the usual repair process observed in p22 phage depends on long matching sequences (typically 50 to 150 bp) through homologous recombination [ 41‒ 45].

Our study further demonstrated that a hotspot region exists for MMEJ in T7 phage, suggesting a preference for site selection during MMEJ events. In addition, we summarized some patterns of repeat sequences that are frequently used for MMEJ. First, relatively longer repeat sequences are more frequently used compared with shorter sequences. In our study, most selected repeated sequences are over 10 bp, with the largest reaching up to 16 bp. Second, repeat sequences with lower GC content were more frequently used than those with higher GC content. In our study, most of the selected repeated sequences have GC contents ranging from 30% to 50%, while more potential repeat sequences with higher GC contents were not selected. This finding is different from previous reports in the bacteriophage T4 [34]. These patterns may provide valuable insights for designing efficient gRNA targeting the T7 genome. Designing gRNA outside the hotspot region can reduce the impact of MMEJ events, and this strategy was applied for the first time in our study compared with previous studies. Furthermore, it was found that point mutations and MMEJ events at target sites are the result of gene expression by T7 phage, instead of *E*. *coli*. Our study indicated that the *1*. *3* gene of the T7 phage is involved in the MMEJ process. A significant decrease in the frequency of MMEJ in the T7 phage was observed after deletion of the *1*. *3* gene, and the efficiency of gene editing in this phage was improved significantly. Compared to genome editing tools based on Cas3 and Cas9, this research achieved higher efficiency of genome editing [ 8, 9]. On the other hand, in comparison with the editing tools based on Cas13a, the operations in this study are more convenient, and it takes less time to obtain positive phages [13].

However, there were still many survived T7 phage escaped the cleavage by DNA repair with point mutations in the PAM and spacer sequence of gene *5*, which is an essential gene for the T7 phage. Therefore, reducing the mutation frequency of PAMs and spacer sequences directly to diminish the escape rate of T7 phages is a challenging task. Thus, designing two gRNAs corresponding to adjacent protospacers and the CRISPR/Cas9 complex will be directed to make two cleavages on the genome, thereby excising the intervening sequence, or designing gRNAs with higher targeting efficiency may be a good option to reduce the escape rate by point mutations of the PAM and spacer sequence [11].

In summary, this study systematically revealed the mechanisms by which the T7 phage escapes from the cleavage of CRISPR/Cas9. By designing an efficient gRNA and deleting the key gene *1*. *3* of the T7 phage, we successfully reduced the escape rate and improved the efficiency of genome editing. This research will serve as a reference for studying the mechanisms of T7 phage escaping cleavage of other Cas effector proteins and other bacteriophages evading CRISPR/Cas systems. This study will contribute to advancing the development of efficient CRISPR/Cas-based genome editing tools for bacteriophages and accelerating the development of bacteriophages for therapeutic purposes.

## Supporting information

supplementaryData

## Supplementary Data

Supplementary data is available at *Acta Biochimica et Biophysica Sinica* online.
