# Supplementary material for: Increasing CRISPR/Cas9-mediated gene editing efficiency in T7 phage by reducing the escape rate based on insight into the survival mechanism: T7 Phage escape in CRISPR/Cas9: strategies for efficient editing
Source: Acta Biochim Biophys Sin (Shanghai). 2024 May 16;56(6):937–44. doi: 10.3724/abbs.2024030 (PMC11294054; doi:10.3724/abbs.2024030)
Supplement: supplementaryData [file supplementaryData.pdf]

## Supplementary Material

### Supplementary Table S1. Plasmids and strains used in this study.

| Strains or plasmids | Description | Source or reference |
|---|---|---|
| **Strains** | | |
| *E. coli* DH5α | Commercial transformation host | GIBCO BRL, Life Technologies |
| *E. coli* MG1655 | Host for testing the escape rate | Lab storage |
| Bacteriophage T7 | T7 Phage for testing the escape rate | Lab storage |
| Bacteriophage T7Δ*1.3* | T7 Phage Δ*1.3* for testing the escape rate | This study |
| **Plasmids** | | |
| pEcCas | Constitutive expression of Cas9 and inducible expression of λ-Red recombination systems | Lab storage |
| pEcgRNA-T7-1.3 | Derived from pEcgRNA, target T7 *1.3* in Bacteriophage T7 | This study |
| pEcgRNA-T7-1.7 | Derived from pEcgRNA, target T7 *1.7* in Bacteriophage T7 | This study |
| pEcgRNA-T7-1.2 | Derived from pEcgRNA, target T7 *1.2* in Bacteriophage T7 | This study |
| pEcgRNA-T7-4.3-original | Derived from pEcgRNA, target T7 *4.3* in Bacteriophage T7 | This study |
| pEcgRNA-T7-4.3-new | Derived from pEcgRNA, target T7 *4.3* another protospacer in Bacteriophage T7 | This study |
| pEcgRNA-T7-1.3-donor | Derived from pEcgRNA, target T7 *1.3* in Bacteriophage T7 with donor DNA | This study |
| pEcgRNA-T7-1.7-donor | Derived from pEcgRNA, target T7 *1.7* in Bacteriophage T7 with donor DNA | This study |
| pEcgRNA-T7-4.3-original-donor | Derived from pEcgRNA, target T7 *4.3* original protospacer in Bacteriophage T7 with donor DNA | This study |
| pEcgRNA-T7-4.3-new-donor | Derived from pEcgRNA, target T7 *4.3* new protospacer in Bacteriophage T7 with donor DNA | This study |

### Supplementary Table S2. Oligonucleotides used in this study.

| Oligos | Sequence (5′→3′) |
|---|---|
| T71.7-HR-upup | cctactcgagttcatgtgcatatcctgacgcttgcgtatattct |
| T71.7-HR-updn | gtggggttgacttgaagttacatcagaacacctccttgatt |
| T71.7-HR-dnup | aatcaaggaggtgttctgatgtaacttcaagtcaaccccac |
| T71.7-HR-dndn | tccccttttgcttatggagcctttatagacaccacgagggttcc |
| T7-1.7-KO-verf-up | gttggtaaattccttgcgg |

| | |
|---|---|
| T7-1.7-KO-verf-dn | tcgaccatacgctggcag |
| T7-1.3-HR-upup | atgattgaccttcttccggt |
| T7-1.3-HR-updn | aaggtgagccagtgtgattacataatgtttatctcctatt |
| T7-1.3-HR-dnup | aataggagataaacattatgtaatcacactggctcacctt |
| T7-1.3-HR-dndn | attgctttctcaaagtcacc |
| T7-1.3-KO-verf-up | attcgcaagagtggcct |
| T7-1.3-KO-verf-dn | gaaccggacgtttcttgtc |
| T7-4.3-HR-upup | gtatccgcttctggtgaatc |
| T7-4.3-HR-updn | atggtttctcctgtttgattacatggtgtgtctcctttagtg |
| T7-4.3-HR-dnup | cactaaaggagacacaccatgtaatcaaacaggagaaaccat |
| T7-4.3-HR-dndn | agtctgtcttgtccaagtc |
| T7-4.3-KO-verf-up | agaccacatctcaatcgtc |
| T7-4.3-KO-verf-dn | ttctggtttcttccagcc |
| gRNA-donor-up | gctccataagcaaaaggggat |
| gRNA-donor-dn | tgcacatgaactcgagtaggga |
| gRNA-donor-verf-up | ttgacagctagctcagtcc |
| gRNA-donor-verf-dn | gatcaccgcttccctcat |
| donor-ce-1 | taaaataaggctagtccgtt |
| donor-ce-2 | gatgacatcagtcgatcatag |
| T7-1.7-gRNA-up | tcctaggtataatactagtCCTTCGATTGTCTCAGCGGAgttttagagctagaaatagc |
| T7-1.3- gRNA-up | tcctaggtataatactagtCGAGGCTGCCCGTGTGGCAAgttttagagctagaaatagc |
| T7-4.3-original-gRNA-up | tcctaggtataatactagtGGCCACACAGTCACGCGCTCgttttagagctagaaatagc |
| T7-4.3-New- gRNA-up | tcctaggtataatactagtATTTGGAAAGCTGTTGAGCTgttttagagctagaaatagc |
| T7-1.2- gRNA-up | tcctaggtataatactagtGTCCTCGTCGTGGTGGTAGAgttttagagctagaaatagc |
| T7-5- gRNA-up | tcctaggtataatactagtAATCAAACGTGACAACACAAgttttagagctagaaatagc |
| gRNA-dn-univers | ctagtattatacctaggactgagct |

Sequences in uppercase represent the guide sequence of gRNA used in genes cutting plasmids construction.

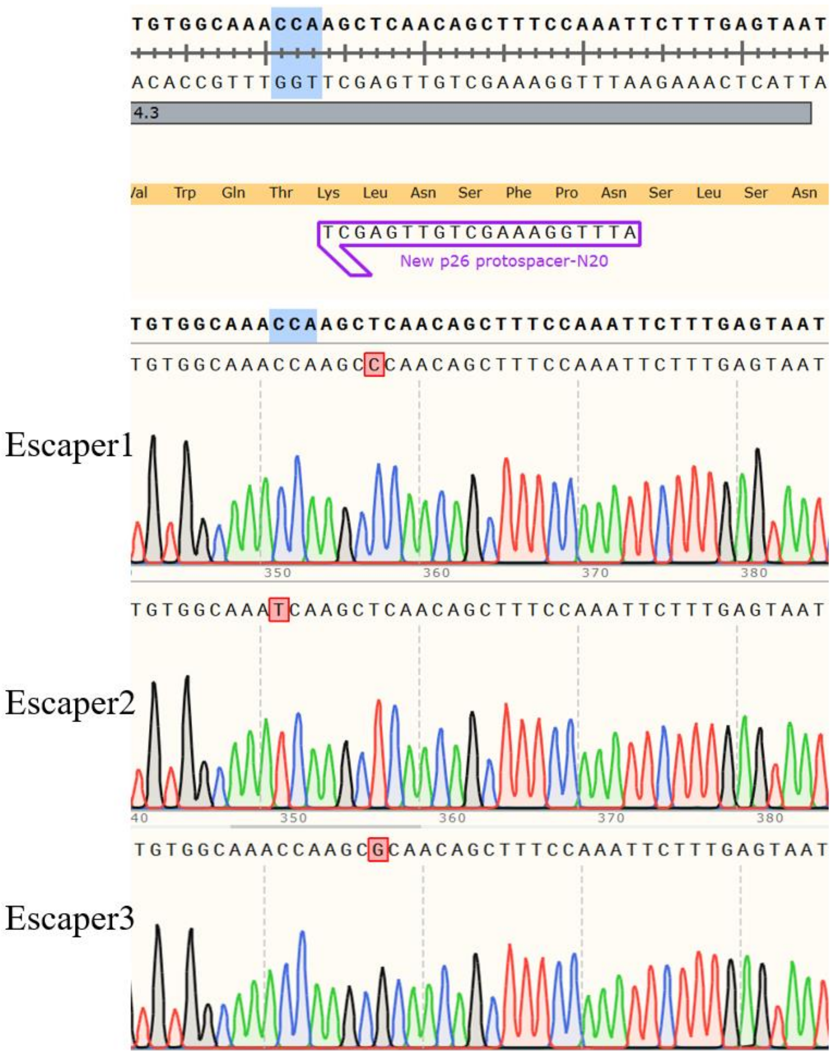


**Supplementary Figure S1. Sequencing results of escaped phage targeted the *4.3* gene with a new site** The purple sequence represents the targeted *4.3* N20 sequence, the blue sequence indicates the PAM region. The three peak profiles show the DNA sequencing results of three randomly selected escaped plaques.

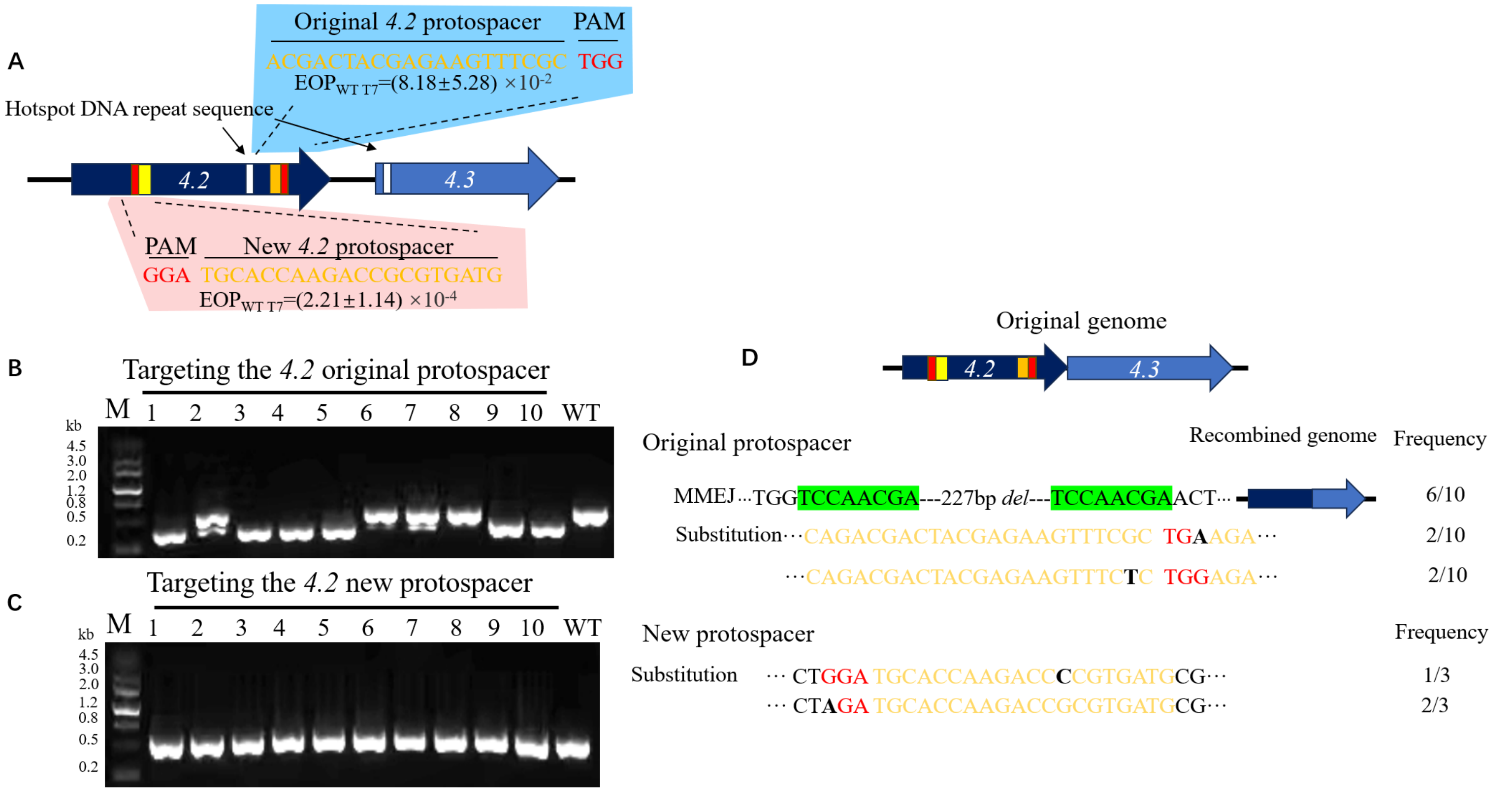


**Supplementary Figure S2. The hotspot DNA region existed for MMEJ events and the design of targeted sites bypassing the hotspot DNA region can reduce MMEJ events and increase the efficiency of gene deletion** (A) The information of the *4.2* targeted sites including original *4.2* protospacer and new *4.2* protospacer, the hotspot DNA region marked in white. (B) PCR amplification results of escaped plaques under the targeting of the original *4.2* site. (C) PCR amplification results of single escaped plaques under the targeting of the new *4.2* site. (D) DNA Mutation type of *4.2* by targeting the original site and the new site. “WT” represents the wild type.

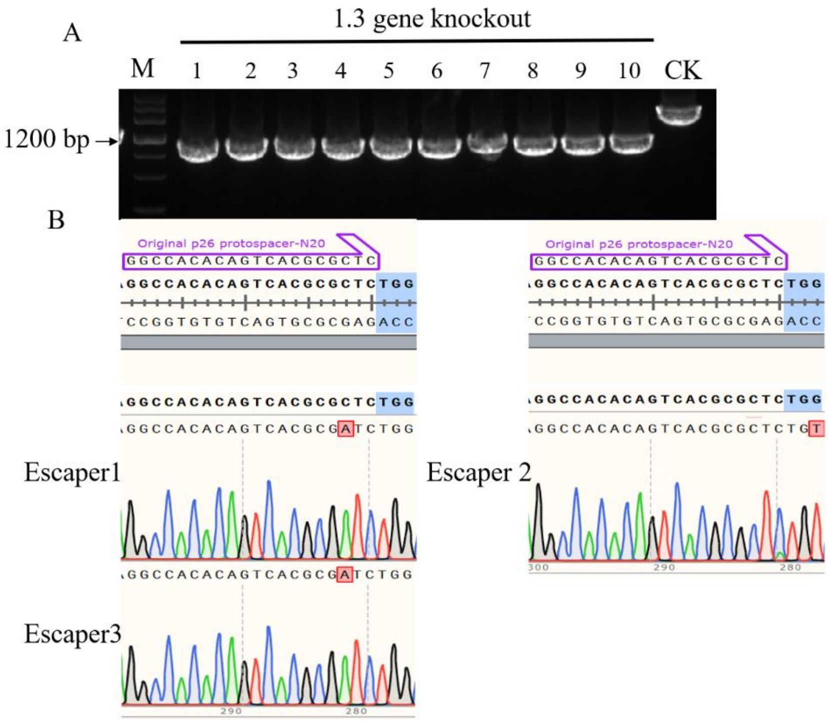


**Supplementary Figure S3. Results of *1.3* gene knocked out in T7 phage and the DNA sequencing results of *4.3* escaped plaques whose DNA amplification results showed the same size as the wild type in T7 Δ*1.3*.** (A) Results of 1.3 gene knocked out in T7 phage and showed that all randomly selected 10 survived plaques were positive, number 8 was chosen randomly for further experiments. (B) Sequencing results of three randomly selected *4.3* escaped plaques which showed the same size as the wild type in T7 Δ*1.3*, showed point mutations at the targeted site or PAM region. "CK" was the PCR amplification of T7 phage without any cleavage.

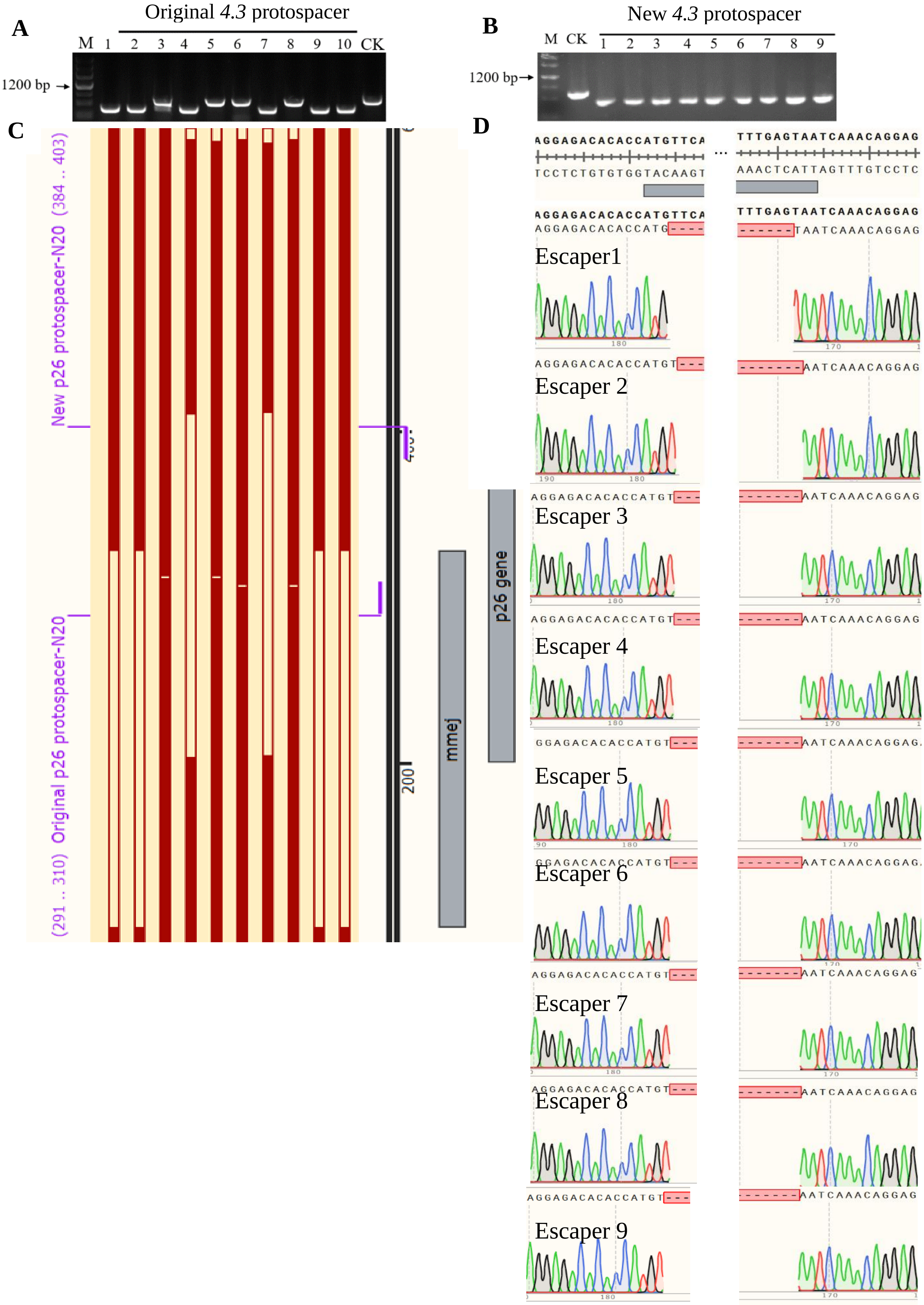
A
Original 4.3 protospacer
M 1 2 3 4 5 6 7 8 9 10 CK
1200 bp
B
New 4.3 protospacer
M CK 1 2 3 4 5 6 7 8 9
1200 bp
C
New p26 protospacer-N20 (384 .. 403)
Original p26 protospacer-N20 (291 .. 310)
200
mmej
p26 gene
D
Escaper1
Escaper 2
Escaper 3
Escaper 4
Escaper 5
Escaper 6
Escaper 7
Escaper 8
Escaper 9

**E**

MMEJ:

Point mutation：

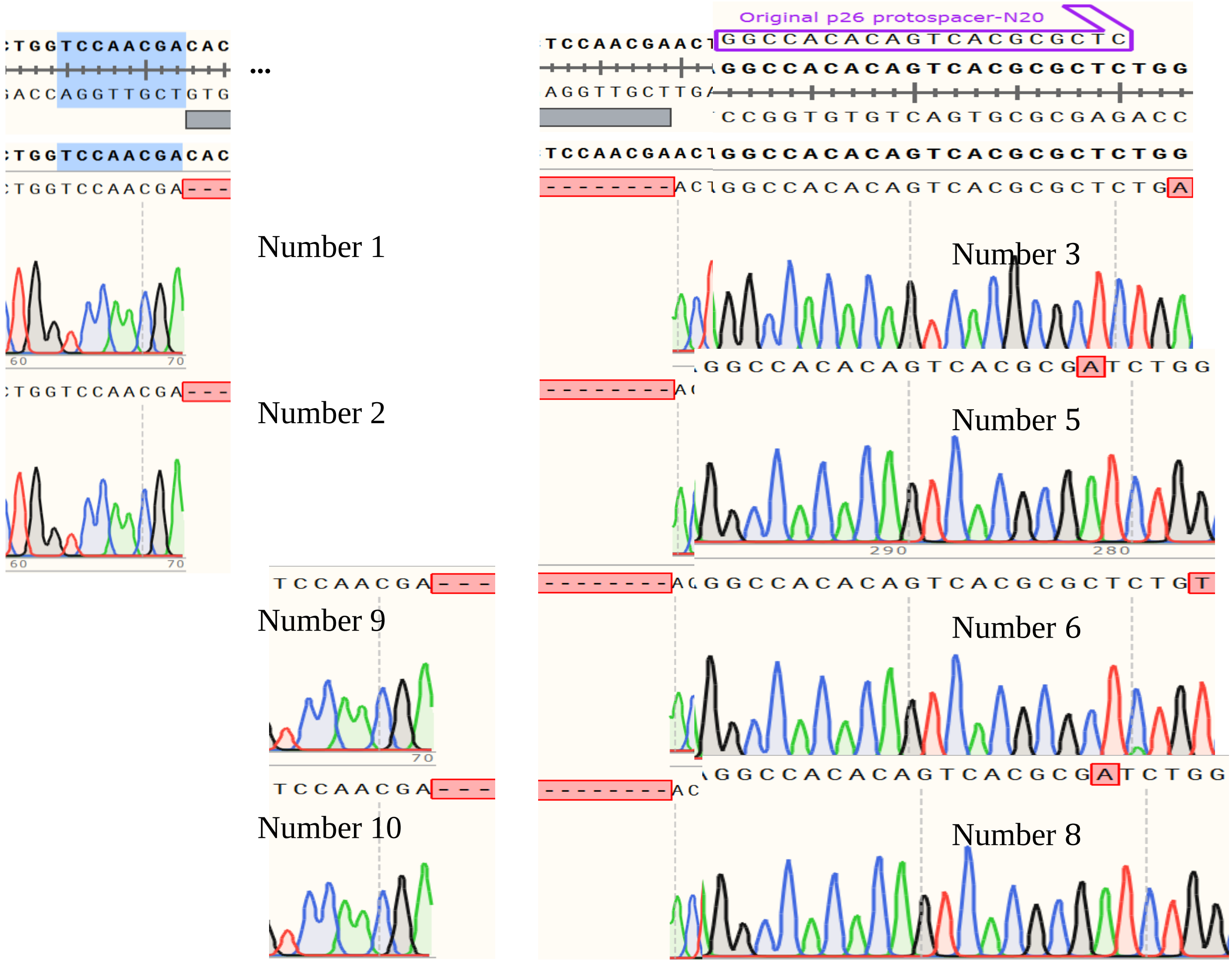


Knocked out：

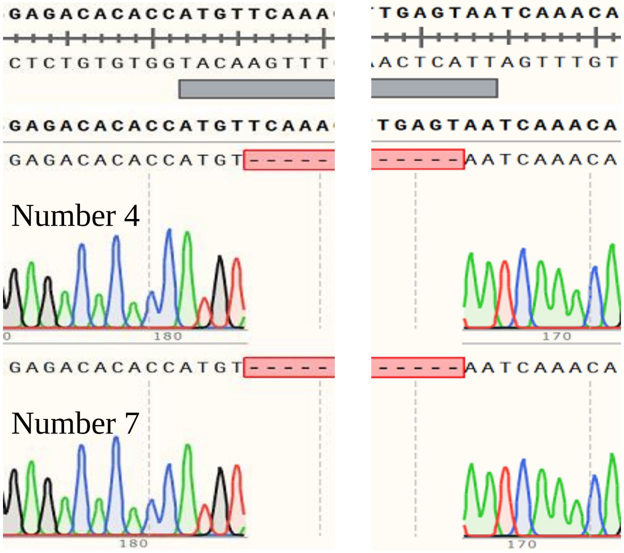

**Supplementary Figure S4. The gene editing results of *4.3* targeting the original site and new site** (A) The results of gene editing targeted the original site of *4.3*. (B) The results of gene editing targeted the new site of *4.3*. (C) Sequencing results of DNA bands showed in (A) were aligned with alignment results corresponding to each lane. The results showed that although there were six DNA bands in (A) that were similar in size to the expected ones, only two of them were *4.3* deleted phages, and the others were escaped phages through MMEJ. (D) Sequencing results of (B) with peak profile and it was showed that all tested phages were *4.3* deleted phages. (E) peak profile of (C). “CK” was the PCR amplification of T7 phage WT.
